# Prior Antiplatelet Therapy and Stroke Risk in Critically Ill Patients Undergoing Extracorporeal Membrane Oxygenation

**DOI:** 10.3390/ijerph18168679

**Published:** 2021-08-17

**Authors:** Tak-Kyu Oh, In-Ae Song, Sol-Yi Lee, Hey-Ran Choi

**Affiliations:** 1 Department of Anesthesiology and Pain Medicine, Seoul National University Bundang Hospital, Seongnam 13620, Korea; airohtak@hotmail.com; 2 Department of Anesthesiology and Pain Medicine, Inje University Seoul Paik Hospital, Seoul 04551, Korea; dlthfdl92@gmail.com

**Keywords:** aspirin, clopidogrel, extracorporeal membrane oxygenation, intensive care units, stroke

## Abstract

We aimed to investigate whether prior exposure to antiplatelet therapy (anti-PLT) was associated with stroke incidence after the initiation of extracorporeal membrane oxygenation (ECMO) therapy. We conducted a population-based cohort study based on health records obtained from the National Health Insurance Service database in South Korea. Adult patients (aged ≥ 18 years) who underwent ECMO therapy in the intensive care unit during 2009–2018 were enrolled. In total, 17,237 patients who underwent ECMO therapy were included; stroke occurred in 779 (4.5%) of 17,237 patients within 7 days of initiating the ECMO therapy. The number of patients in the anti-PLT and control groups was 3909 (22.7%) and 13,328 (77.3%), respectively. In the multivariable logistic regression analysis, the anti-PLT group showed 33% lower incidence of stroke than the control group (odds ratio (OR): 0.67, 95% confidence interval (CI): 0.55–0.82; *p* < 0.001). The cardiovascular group showed 35% lower incidence of stroke than the control group (OR: 0.65, 95% CI: 0.52–0.78; *p* < 0.001), whereas the respiratory group (*p* = 0.821) and the other group (*p* = 0.705) did not show any significant association. Prior anti-PLT therapy was associated with a lower incidence of stroke within 7 days of initiating ECMO therapy, which was more evident in the cardiovascular group.

## 1. Introduction

To treat patients with refractory cardiac and/or respiratory failure, extracorporeal membrane oxygenation (ECMO) has been used as an option of rescue therapy [1,2]. The clinical indications of ECMO support include post-cardiac surgery management, heart failure, intractable arrhythmia, heart inflammation, pulmonary hypertension, severe trauma, respiratory failure, and acute respiratory distress syndrome (ARDS) [3,4,5,6,7]. In South Korea, 21,129 ECMO procedures were conducted in adult patients from 2005 to 2018, and the preference for ECMO therapy continuously increased [8].

Initiation of extracorporeal circulation stimulates inflammation and activates the coagulation cascade, leading to thrombosis of the ECMO circuit and the occurrence of thromboembolic complications after ECMO therapy [9]. A recent study reported a 100% occurrence of venous thromboembolism among 13 patients with coronavirus disease (COVID-19)-related ARDS who underwent venovenous (VV) ECMO therapy [10]. Although the clinical use of ECMO therapy as a life-saving therapy has been expanding [11], the systemic inflammatory response to extracorporeal circulation triggers various complications [12]. One of the most severe complications in critically ill patients undergoing ECMO therapy is stroke [13,14,15]. Moreover, bleeding is a frequent complication associated with ECMO therapy [16], and hemorrhagic stroke has also been frequently reported in patients undergoing ECMO therapy [13].

Thus, risk evaluation and prevention of stroke in patients undergoing ECMO are critical safety issues [17]. Antiplatelet (anti-PLT) drugs (aspirin and clopidogrel) have been used in monotherapy or in combination to prevent acute cardiac or vascular events [18]. They inhibit the coagulation effect of platelets to prevent the formation of blood clots, hence protecting the patients from fatal vascular complications such as stroke [19]. Because patients who undergo ECMO therapy have a higher risk of stroke [17], prior use of anti-PLT drugs might affect the risk of stroke after initiating ECMO therapy. However, to the best of our knowledge, no study has evaluated the impact of prior anti-PLT drug therapy on the incidence of stroke among critically ill patients undergoing ECMO.

Therefore, we aimed to investigate whether prior anti-PLT therapy was associated with the incidence of stroke after initiating ECMO therapy. We hypothesized that prior anti-PLT therapy might decrease the risk of acute ischemic stroke but increase the risk of acute hemorrhagic stroke. 

## 2. Materials and Methods

### 2.1. Study Design and Ethical Statement

In this population-based cohort study, the guidelines for Reporting of Observational Studies in Epidemiology were followed [20]. The Institutional Review Board of Seoul National University Bundang Hospital (X-2001-586-902) and the Health Insurance Review and Assessment Service approved the study protocol. The need for informed consent was waived because analyses were performed retrospectively with anonymized data, which were derived from the South Korean National Health Insurance Service (NHIS) database.

### 2.2. NHIS Database and Study Population

In this study, the health records were extracted from the NHIS database. As the sole public health insurance system, information regarding all disease diagnoses and prescriptions corresponding to drugs and/or procedures had to be registered in the NHIS database by physicians for patients to receive financial support for any diagnostic test and treatment charges in South Korea. This study included all adult patients (aged ≥ 18 years old) who received ECMO support in the intensive care unit (ICU; outside the operating room) after hospitalization from 2009 to 2018 (10 years). The cases of Novalung therapy were not considered for ECMO in this study. For patients who received two or more episodes of ECMO therapy, only the first episode of ECMO therapy was considered in this study. Additionally, cases of ECMO therapy for stroke treatment were excluded from the analysis because we focused on the newly developed stroke after initiating ECMO. As the NHIS did not provide distinguishable prescription codes for venoarterial (VA) and VV ECMO, we classified all ECMO patients into three groups using main diagnosis at ECMO therapy: (1) cardiovascular group (cardiovascular disease, shock, or post-cardiac arrest), (2) respiratory group (ARDS or respiratory failure), and (3) other group. The NHIS contains the records of all diagnoses made during hospitalization; the diagnosis registered as the primary morbidity of treatment was then classified as the main diagnosis.

### 2.3. Exposure (Aspirin and Clopidogrel Use)

We defined exposure to anti-PLT drugs (aspirin and clopidogrel) based on the prescription medication from the hospital or outpatient clinic in the NHIS database. The anti-PLT group included ECMO patients who were prescribed aspirin or clopidogrel continuously for at least 1 month before starting ECMO therapy, and the control group included the remaining patients. Therefore, even if they were prescribed anti-PLT drugs in the past, they were considered as the control group if they did not take aspirin 1 month before starting ECMO therapy. The anti-PLT therapy was continued during hospitalization in almost all the patients in the anti-PLT group who could take it orally or through the enteral route, using the Levin tube. However, anti-PLT drug use was sometimes interrupted in patients who could not take it through the oral or enteral routes.

### 2.4. Endpoints (Stroke)

The primary endpoint was the development of stroke within 7 days of initiating ECMO therapy. A case of stroke among the ECMO patients was identified by the codes of the International Classification of Diseases, 10th revision (ICD-10) (I60–I63) following diagnostic brain imaging using computed tomography or magnetic resonance imaging. Particularly, stroke was divided as hemorrhagic stroke (I60, I61, and I62) and ischemic stroke (I63). In South Korea, the ICD-10 codes of stroke are registered in the NHIS database by a physician for financial coverage of treatment or brain imaging test after the diagnosis of stroke.

### 2.5. Confounders

The following factors were considered as confounders: demographic information (age and sex), socioeconomic status-related information (area of residence and annual income level during ECMO therapy), and Charlson comorbidity index scores that were calculated using the registered ICD-10 codes assigned one year before the start date of ECMO therapy (Table S1). In addition, information on the following treatment-related variables was collected: length of hospital stay (days) and duration of ECMO therapy (days). The annual volume of cases involving ECMO treatment was examined to determine the ability of the ECMO centers in South Korea to administer treatment because this factor influences the outcomes of ECMO treatment [21]. It was divided into four groups using quartile ratios (Q1, <17; Q2, 17–36; Q3, 37–80; and Q4, >81). Finally, the prior use of novel oral anticoagulants (NOAC, such as apixaban, edoxaban, rivaroxaban, and dabigatran) and the use of low-molecular-weight heparin during hospitalization were investigated and considered as confounders because they can affect anticoagulation in critically ill patients who underwent ECMO.

### 2.6. Statistical Analysis

The clinicoepidemiological characteristics of patients who underwent ECMO are presented as mean values with standard deviation (SD) for continuous variables and as numbers with percentages for categorical variables. First, we fitted the data using a multivariable logistic regression model for the development of stroke in the entire ECMO cohort. All the covariates were included in the multivariable model for adjustment; however, the Charlson comorbidity index was included in a multivariable model separate from other underlying diseases used to calculate the Charlson comorbidity index to avoid multicollinearity within the model. Second, we performed subgroup analyses by constructing another multivariable logistic regression model, and the anti-PLT group was divided into three groups: aspirin monotherapy group, clopidogrel monotherapy group, and dual anti-PLT group. Third, we fitted the data using a multivariable logistic regression analysis for ischemic and hemorrhagic stroke among all ECMO patients to examine whether prior anti-PLT drug use affected the different types of stroke. Fourth, we performed subgroup analyses according to the main diagnosis at ECMO therapy considering the ECMO type (cardiovascular group, respiratory group, and other group). Fifth, we fitted the data using a multivariable logistic regression model for the development of stroke among ECMO patients who survived for ≥7 days because some ECMO patients did not experience stroke because of earlier mortality before 7 days after initiating ECMO therapy. Sixth, we fitted the data using a multivariable logistic regression analysis for the development of stroke within 30 days (contrary to the 7 days in the main analysis) to examine whether these associations would differ if the duration of stroke evaluation was prolonged to 30 days after initiating ECMO therapy. Lastly, to enhance the robustness of our findings, we performed 1:1 propensity score (PS) matching between the anti-PLT and control groups to reduce the risk of bias [22]. The nearest neighbor method was used without replacement and with a caliper of 0.25 for the PS matching. All covariates were included in the PS model, and a logistic regression analysis was performed to calculate the PSs. The absolute value of the standardized mean difference (ASD) was used to evaluate the balance between the groups before and after PS matching. The ASD was set at <0.1 to confirm adequate balance between the two groups, while t-test and chi-square test were used for comparing continuous variables and categorical variables, respectively. After confirming adequate balance between the two groups, we performed a logistic regression analysis for the development of stroke in the PS-matched cohort. The results of the logistic regression are presented as odds ratios (ORs) with 95% confidence intervals (CIs). We confirmed that there was no multicollinearity in all the multivariable models of the entire ECMO cohort with a variance inflation factor of <2.0. The Hosmer–Lemeshow test was used to test the goodness of fit of the multivariable models. Additionally, receiver operating characteristic (ROC) curve analysis was performed to identify the validity of the multivariable model for the development of stroke. All statistical analyses were performed using R software (version 4.0.3 with R packages, the R Project for Statistical Computing, Vienna, Austria). *p* < 0.05 was considered statistically significant.

## 3. Results

### 3.1. Study Population

**Figure 1:**
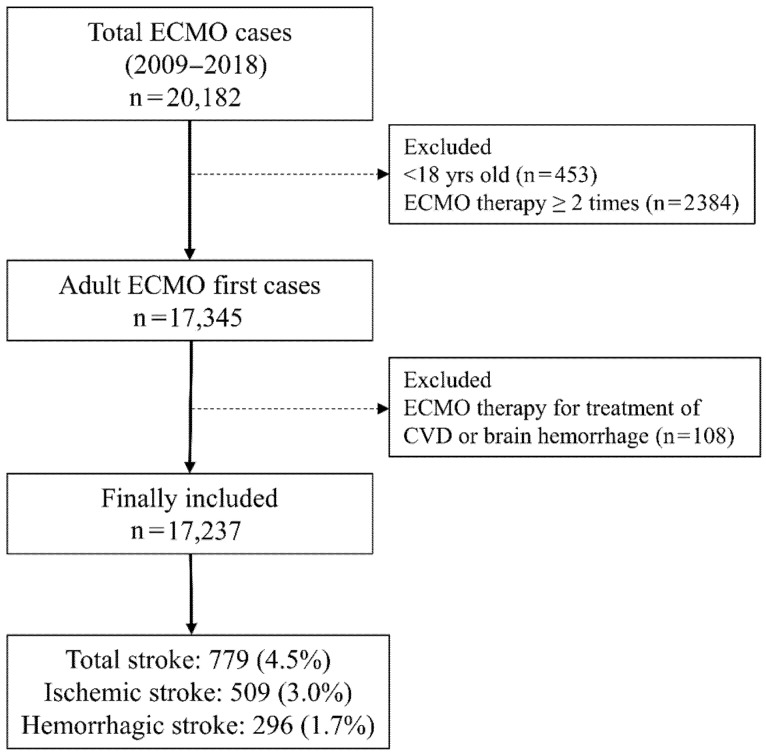
Flowchart depicting the patient selection process. ECMO, extracorporeal membrane oxygenation; CVD, cardiovascular diseases.

Between 1 January 2009 and 31 December 2018, a total of 20,182 ECMO cases were registered in 127 hospitals in South Korea. After excluding 453 pediatric patients aged < 18 years and 2384 patients who received ECMO therapy for two or more times during the study period, a total of 17,345 adult patients were initially screened. Next, 108 patients who received ECMO therapy for stroke treatment were excluded, leaving a final sample of 17,237 ECMO patients. Among them, stroke occurred in 779 ECMO patients (4.5%) within 7 days of initiating ECMO therapy. There were 509 (3.0%) cases of ischemic stroke and 296 (1.7%) cases of hemorrhagic stroke; 26 patients (0.2%) experienced both ischemic and hemorrhagic stroke within 7 days of initiating ECMO therapy (Figure 1). The clinicoepidemiological characteristics of the patients are presented in Table 1. The mean duration of brain imaging from the date of starting ECMO therapy among ECMO patients with stroke was 2.4 (SD: 3.2) days. Among all the ECMO patients, the anti-PLT group had 3909 (22.7%) patients and the control group had 13,328 (77.3%) patients.

### 3.2. Incidence of Stroke in the Entire Cohort

Table 2 shows the results of the multivariable logistic regression model for the development of stroke in the entire cohort. In the multivariable model, the anti-PLT group showed a 33% lower incidence of stroke than the control group (OR: 0.67, 95% CI: 0.55–0.82; *p* < 0.001; Model 1). In addition, the anti-PLT group showed 37% (OR: 0.73, 95% CI: 0.55–0.92; *p* < 0.001; Model 2) and 44% (OR: 0.56, 95% CI: 0.40–0.80; *p* < 0.001; Model 3) lower incidences of ischemic and hemorrhagic stroke than the control group, respectively. In the subgroup analysis (Model 4), the aspirin monotherapy, clopidogrel monotherapy, and dual anti-PLT therapy groups showed 25% (OR: 0.75, 95% CI: 0.59–0.97; *p* = 0.025), 45% (OR: 0.55, 95% CI: 0.45–0.80; *p* = 0.007), and 37% (OR: 0.63, 95% CI: 0.44–0.88; *p* = 0.007) lower incidences of stroke, respectively, compared to the control group. Table S2 shows the results of the multivariable logistic regression model for stroke among ECMO patients who survived for ≥7 days (*n* = 11,975). Compared to the control group, the incidences of total stroke and ischemic stroke in the anti-PLT group were 26% lower (OR: 0.74, 95% CI: 0.58–0.95; *p* = 0.026) and 24% (OR: 0.78, 95% CI: 0.59–0.99; *p* = 0.048), respectively; the incidence of hemorrhagic stroke was not significantly different between the two groups (*p* = 0.238). Table S3 shows the results of the multivariable logistic regression analysis for stroke within 30 days (contrary to the 7 days in the main analysis) among all ECMO patients. Compared to the control group, the incidence of total stroke within 30 days in the anti-PLT group was 30% lower (OR: 0.70, 95% CI: 0.57–0.85; *p* < 0.001) than that in the control group.

### 3.3. Subgroup Analyses According to Main Diagnosis at ECMO Therapy

Table 3 shows the results of the subgroup analysis according to main diagnosis at ECMO therapy, considering the ECMO type. In the cardiovascular group, the anti-PLT group shows 35% (OR: 0.65, 95% CI: 0.52–0.78; *p <* 0.001), 30% (OR: 0.70, 95% CI: 0.55–0.90; *p* = 0.006), and 50% (OR: 0.50, 95% CI: 0.35–0.75; *p* < 0.001) lower incidences of total, ischemic, and hemorrhagic stroke than the control group, respectively. However, the anti-PLT group in both the respiratory group and the other group did not show a significant association for total, ischemic, and hemorrhagic stroke (all *p* > 0.05).

### 3.4. Sensitivity Analysis after PS Matching

Table 4 shows the results of the comparison of clinicoepidemiological characteristics between the anti-PLT and control groups before and after PS matching. Before PS matching, mean patient age in the anti-PLT group was 67.1 (SD: 10.9) years, which was higher than that of 57.2 (SD: 15.2) years in the control group (*p* < 0.001). After 1:1 PS matching, a total of 7818 patients who received ECMO (3909 in each group) were included in the sensitivity analysis. All the ASDs between the two groups were below 0.1 after PS matching, indicating that an adequate balance was achieved between the two groups. Table 5 shows the results of stroke incidence before and after PS matching. After PS matching, the incidences of total stroke in the anti-PLT and control groups were 4.4% (171/3909) and 6.0% (233/3909), respectively. In the logistic regression analysis of the PS-matched cohort, the anti-PLT group showed a 25% lower incidence of stroke than the control group (OR: 0.75, 95% CI: 0.60–0.90; *p* = 0.009). In addition, the anti-PLT group showed a 21% lower incidence of ischemic stroke (OR: 0.79, 95% CI: 0.60–0.98; *p* = 0.043) and a 35% lower incidence of hemorrhagic stroke (OR: 0.65, 95% CI: 0.50–0.94; *p* = 0.020) than the control group.

## 4. Discussion

This population-based cohort study showed that prior anti-PLT therapy was associated with a lower incidence of stroke within 7 days of initiating ECMO therapy. Contrary to our hypothesis, the incidence of both ischemic and hemorrhagic stroke decreased in the anti-PLT group. However, in sensitivity analysis of ECMO patients who survived for ≥7 days, only ischemic stroke decreased in the anti-PLT group. Moreover, this association was most significant in the cardiovascular group than in the respiratory group and the other group. Our findings suggest that anti-PLT therapy can be a useful option in preventing stroke among patients undergoing ECMO in the ICU.

Using nationwide cohort claim data of South Korea, we reported a 4.5% incidence of total stroke among ECMO patients during the 7-day period after initiating ECMO therapy. The incidences of ischemic stroke, hemorrhagic stroke, and both ischemic and hemorrhagic stroke were 3.0%, 1.7%, and 0.2%, respectively. Previous retrospective cohort studies in single centers reported that the incidence of ischemic stroke ranged from 5.3% to 16.3% in patients undergoing ECMO [13,14,15,23]. By contrast, the incidence of hemorrhagic stroke ranged from 2.8% to 21% [13,24,25]. These previous studies used data obtained from single centers [13,14,15,23,24,25], whereas we obtained data from nationwide claims data. However, it was sometimes difficult to test critically ill patients requiring ECMO therapy for the presence of stroke because of hemodynamic instability. Thus, the actual incidence of stroke might have been underestimated in this study.

We focused on the development of stroke within 7 days of initiating ECMO therapy because of the recovery period of platelet function after discontinuation among ECMO patients in the anti-PLT group. Our results suggest that prior anti-PLT therapy might modify the risk of stroke during the 7-day period after initiating ECMO therapy by inhibiting blood clots and emboli formation [19]. Critically ill patients who undergo ECMO therapy require monitoring of coagulation function, and sometimes anticoagulation therapy is needed to prevent the development of stroke [26]. Although we did not evaluate the effect of coagulation function or anticoagulation on the risk of stroke among ECMO patients, it is possible that ECMO patients who had received anti-PLT therapy might have a lower risk of stroke than those who did not receive the anti-PLT therapy. 

Interestingly, our study showed that a significant association between prior anti-PLT therapy and the occurrence of stroke was observed only in the cardiovascular group and not in the respiratory group or the other group. The cardiovascular group had a higher possibility of receiving VA ECMO than the respiratory group. In a recent cohort study, patients who underwent ECMO therapy with a primary diagnosis of acute respiratory failure constituted the VV ECMO group, whereas patients who underwent ECMO therapy with a primary diagnosis of cardiogenic shock or cardiac arrest constituted the VA ECMO group [27]. Furthermore, a recent study reported that 98% of ARDS patients associated with COVID-19 underwent VV ECMO therapy, whereas only 2% underwent VA ECMO therapy [28]. As recent studies reported that VA ECMO therapy is an independent predictor for a higher prevalence of ischemic stroke and hemorrhagic stroke [13,29], the impact of prior anti-PLT therapy might be more important in critically ill patients who underwent VA ECMO. However, we did not identify the ECMO type according to prescription codes, and this is a limitation of the present study; further studies are needed regarding this issue. Moreover, some confounding factors, other than ECMO itself, such as characteristics of the cardiovascular group might have played a role in the causation of stroke. Therefore, further study on the impact of prior anti-PLT therapy on ECMO patients considering the ECMO type is needed.

Among the covariates listed in Table 2, prior NOAC use, such as apixaban, edoxaban, rivaroxaban, and dabigatran, was associated with a 70% lower incidence of stroke among total ECMO patients in this study. NOAC use has been recommended for reducing the incidence of stroke [30]. However, the direct effect of NOAC use on reducing ischemic and hemorrhagic stroke, compared to anti-PLT drugs such as aspirin and clopidogrel, has not been identified clearly [31]. Therefore, the clinical efficacy of NOACs in preventing stroke among critically ill patients who underwent ECMO should be evaluated in the future.

Notably, the results regarding the protective effect of anti-PLT therapy against hemorrhagic stroke should be interpreted carefully because bleeding is one of the complications of anti-PLT therapy [32]. First, the pathophysiology of hemorrhagic stroke among ECMO patients is multifactorial; therefore, the impact of anti-PLT therapy on the risk of bleeding might not be an important factor [24]. Other pre-ECMO parameters, such as sequential organ failure assessment (SOFA) scores, septic shock, and platelet count, might affect the risk of hemorrhagic stroke [24]. Moreover, a rapid decrease in the partial pressure of carbon dioxide after initiating ECMO therapy can cause significant changes in cerebral blood flow because of vascular smooth muscle cell vasoconstriction, which can result in hemorrhagic stroke [33]. Second, the results of the sensitivity analysis of ECMO patients who survived for ≥7 days showed no significant association between prior anti-PLT therapy and the development of hemorrhagic stroke. A previous study reported that hemorrhagic stroke after ECMO therapy significantly increased the mortality of ECMO patients, whereas ischemic stroke was not associated with mortality [13]. This suggests that early mortality of ECMO patients might have affected the incidence of hemorrhagic stroke in our study because some ECMO patients who died due to hemorrhagic stroke had not been diagnosed with hemorrhagic stroke. 

Our study has several limitations. First, some important variables, such as body mass index and lifestyle factors including history of smoking and alcohol use, were not included in this study because the NHIS database did not have a record of these data. Second, the disease severity of the patients who underwent ECMO was not evaluated and confirmed through objective methods such as the Acute Physiology and Chronic Health Disease Classification System II and SOFA scores. Third, as mentioned above, we did not distinguish between VA ECMO and VV ECMO because of limitations associated with the prescription codes for ECMO in South Korea. Therefore, it is impossible to know the proportions of patients who underwent VA ECMO and VV ECMO accurately. Fourth, the generalizability of our findings might have been limited because there were disparities between the Asian and non-Asian populations regarding risk factors for stroke [34]. Fifth, we also did not consider the incidence of ischemic stroke converting to hemorrhagic stroke. Twenty-six patients (0.2%) who experienced both ischemic and hemorrhagic stroke might have had the possibility of hemorrhagic conversion of ischemic stroke, but this issue cannot be identified accurately in this study. Sixth, there might have been some underdiagnosed stroke cases because of the use of neuromuscular blockers and sedatives, which might have affected our results. Lastly, we did not monitor coagulation function, platelet count, and anticoagulation effect among patients undergoing ECMO therapy. These three factors can influence the incidence of complications such as stroke.

## 5. Conclusions

In this population-based cohort study, we have shown that prior anti-PLT therapy (aspirin or clopidogrel) is associated with a lower incidence of stroke within 7 days of initiating ECMO therapy. This association was more evident in the cardiovascular group than in the respiratory group or in the other group. Therefore, prior anti-PLT therapy may not affect the risk of stroke in COVID-19 patients who underwent VV ECMO, and further study is needed to validate this finding. Although this association was observed in both ischemic and hemorrhagic stroke, the impact of anti-PLT therapy on hemorrhagic stroke should be carefully interpreted because it was not evident in ECMO patients who survived ≥7 days. Because of the retrospective study design, which is a limitation, further studies are needed to confirm these findings.

## Figures and Tables

**Table 1:** Clinicoepidemiological characteristics of patients undergoing ECMO.

Variable	Number (%)	Mean (SD)
Age, year		59.5 (15.0)
Sex, male	11,474 (66.6)	
Residence at ECMO therapy		
Capital city (Seoul)	3998 (23.2)	
Other metropolitan city	4004 (23.2)	
Other area	9235 (53.6)	
Year of ECMO therapy		
2009–2010	1568 (9.1)	
2011–2012	2736 (15.9)	
2013–2014	3763 (21.8)	
2015–2016	4234 (24.6)	
2017–2018	4936 (28.6)	
Annual income level at ECMO therapy		
Q1 (Lowest)	4318 (25.1)	
Q2	3142 (18.2)	
Q3	3954 (22.9)	
Q4 (Highest)	5823 (33.8)	
Annual case volume of ECMO therapy		
Q1: <17	4472 (25.9)	
Q2: 17–36	4650 (27.0)	
Q3: 37–80	4725 (27.4)	
Q4: >81	3390 (19.7)	
Length of hospital stay, day		17.2 (15.9)
Duration of ECMO therapy, day		6.2 (8.8)
Prior anti-PLT use	3909 (22.7)	
Prior NOAC use	268 (1.6)	
Prior statin use	3792 (22.0)	
Prior warfarin use	206 (1.2)	
LMWH use during hospitalization	1363 (7.9)	
Charlson comorbidity index		4.1 (2.9)
Hypertension, complicated	2481 (14.4)	
Hypertension, uncomplicated	11,364 (65.9)	
Myocardial infarction	5893 (34.2)	
Congestive heart failure	7012 (40.7)	
Peripheral vascular disease	3635 (21.1)	
Cerebrovascular disease	3281 (19.0)	
Dementia	728 (4.2)	
Chronic pulmonary disease	7075 (41.0)	
Rheumatic disease	1150 (6.7)	
Peptic ulcer disease	5326 (30.9)	
Mild liver disease	6054 (35.1)	
Diabetes without chronic complication	8205 (47.6)	
Diabetes with chronic complication	2592 (15.0)	
Hemiplegia or paraplegia	416 (2.4)	
Renal disease	1759 (10.2)	
Any malignancy	3083 (17.9)	
Moderate or severe liver disease	1208 (7.0)	
Metastatic solid tumor	597 (3.5)	
AIDS/HIV	28 (0.2)	
Dyslipidemia	8355 (48.5)	
Main diagnosis at ECMO therapy		
Cardiovascular group	11,029 (64.0)	
Respiratory group	2003 (11.6)	
Other group	4205 (24.4)	

ECMO, extracorporeal membrane oxygenation; SD, standard deviation; NOAC, novel oral anticoagulant; LMWH, low-molecular-weight heparin; AIDS, acquired immune deficiency syndrome; HIV, human immunodeficiency virus.

**Table 2:** Multivariable logistic regression model for stroke development in the entire cohort.

Variable	Multivariable Model	*p*-Value
	Odds Ratio (95% CI)	
Anti-PLT group for total stroke (vs. control; model 1)	0.67 (0.55, 0.82)	<0.001
Anti-PLT group for ischemic stroke (vs. control; model 2)	0.73 (0.55, 0.92)	0.004
Anti-PLT group for hemorrhagic stroke (vs. control; model 3)	0.56 (0.40, 0.80)	0.001
Subgroup analysis for total stroke (model 4)		
Control (*n* = 13,328)	1	
Aspirin monotherapy (*n* = 2375)	0.75 (0.59, 0.97)	0.025
Clopidogrel monotherapy (*n* = 588)	0.55 (0.45, 0.80)	0.004
Dual anti-PLT therapy (*n* = 946)	0.63 (0.44, 0.88)	0.007
Other covariates in model 1		
Age, year	0.98 (0.97, 0.98)	<0.001
Sex, male (vs. female)	0.96 (0.80, 1.13)	0.631
Residence at ECMO therapy		
Capital city (Seoul)	1	
Other metropolitan city	0.85 (0.65, 1.12)	0.264
Other area	0.96 (0.79, 1.18)	0.706
Year of ECMO therapy		
2009–2010	1	
2011–2012	0.97 (0.70, 1.31)	0.778
2013–2014	0.90 (0.66, 1.24)	0.425
2015–2016	0.98 (0.73, 1.35)	0.9154
2017–2018	1.25 (0.92, 1.70)	0.180
Annual income level at ECMO therapy		
Q1 (Lowest)	1	
Q2	1.11 (0.84, 1.42)	0.477
Q3	1.13 (0.90, 1.44)	0.271
Q4 (Highest)	0.96 (0.78, 1.18)	0.669
Annual case volume of ECMO therapy		
Q1: <17	1	
Q2: 17–36	1.28 (1.02, 1.58)	0.030
Q3: 37–80	0.89 (0.70, 1.14)	0.312
Q4: >81	0.71 (0.53, 0.92)	0.010
Duration of ECMO therapy, day	1.00 (1.00, 1.03)	0.225
Prior NOAC use	0.35 (0.15, 0.73)	0.007
Prior statin use	0.85 (0.60, 1.25)	0.212
Prior warfarin use	1.21 (0.85, 1.45)	0.102
LMWH use during hospitalization	1.06 (0.77, 1.45)	0.642
Charlson comorbidity index, point	1.15 (1.12, 1.20)	<0.001
Hypertension, complicated	0.74 (0.58, 0.95)	0.020
Hypertension, uncomplicated	0.73 (0.59, 0.88)	0.003
Myocardial infarction	1.04 (0.87., 1.27)	0.693
Congestive heart failure	1.04 (0.87, 1.23)	0.658
Peripheral vascular disease	0.82 (0.68, 1.00)	0.053
Cerebrovascular disease	75.11 (58.75, 96.03)	<0.001
Dementia	0.44 (0.30, 0.63)	<0.001
Chronic pulmonary disease	0.83 (0.69, 0.99)	0.035
Rheumatic disease	1.02 (0.73, 1.43)	0.915
Peptic ulcer disease	0.84 (0.70, 1.01)	0.059
Mild liver disease	0.83 (0.69, 0.99)	0.034
Diabetes without chronic complication	0.81 (0.68, 0.97)	0.018
Diabetes with chronic complication	0.73 (0.57, 0.93)	0.009
Hemiplegia or paraplegia	1.16 (0.82, 1.64)	0.391
Renal disease	0.87 (0.66, 1.16)	0.343
Any malignancy	0.94 (0.73, 1.20)	0.594
Moderate or severe liver disease	1.56 (1.13, 2.15)	0.006
Metastatic solid tumor	0.92 (0.53, 1.60)	0.771
AIDS/HIV	0.00 (0.00, -)	0.998
Dyslipidemia	0.87 (0.73, 1.04)	0.114
Main diagnosis at ECMO therapy		
Cardiovascular group	1	
Respiratory group	0.58 (0.42, 0.80)	<0.001
Other	0.57 (0.43, 0.73)	<0.001

AUC: 0.89 (95% CI: 0.88–0.89), Hosmer–Lemeshow test, chi-square: 12.22, df = 8, *p* = 0.141 (Model 1) and chi-square: 13.01, df = 8, *p* = 0.128 (Model 2), CI, confidence interval; ECMO, extracorporeal membrane oxygenation; NOAC, novel oral anticoagulants; LMWH, low-molecular-weight heparin; AIDS, acquired immune deficiency syndrome; HIV, human immunodeficiency virus; AUC, area under the curve.

**Table 3:** Subgroup analyses according to main diagnosis at ECMO therapy considering ECMO type.

Variable	Multivariable Model	*p*-Value
	OR (95% CI)	
Cardiovascular group		
Total stroke		
Anti-PLT group (vs. control)	0.65 (0.52, 0.78)	<0.001
Ischemic stroke		
Anti-PLT group (vs. control)	0.70 (0.55, 0.90)	0.006
Hemorrhagic stroke		
Anti-PLT group (vs. control)	0.50 (0.35, 0.75)	<0.001
Respiratory group		
Total stroke		
Anti-PLT group (vs. control)	0.90 (0.41, 2.11)	0.821
Ischemic stroke		
Anti-PLT group (vs. control)	0.40 (0.10, 1.53)	0.179
Hemorrhagic stroke		
Anti-PLT group (vs. control)	1.42 (0.43, 4.18)	0.745
Other group		
Total stroke		
Anti-PLT group (vs. control)	0.85 (0.48, 1.70)	0.705
Ischemic stroke		
Anti-PLT group (vs. control)	0.95 (0.49, 2.00)	0.883
Hemorrhagic stroke		
Anti-PLT group (vs. control)	0.80 (0.30, 2.18)	0.642

ECMO, extracorporeal membrane oxygenation; OR, odds ratio; CI, confidence interval; PLT, platelet.

**Table 4:** Comparison of clinicoepidemiological characteristics between the anti-PLT and control groups before and after PS matching.

Variable	Entire Cohort (*n* = 17,237)		ASD	*p*-Value	PS-Matched Cohort (*n* = 7818)		ASD	*p*-Value
	Anti-PLT *n* = 3909	Control *n* = 13,328			Anti-PLT *n* = 3909	Control *n* = 3909		
Age, year	67.1 (10.9)	57.2 (15.2)	0.906	<0.001	67.1 (10.9)	66.4 (11.8)	0.061	0.009
Sex, male	2705 (69.2)	8778 (65.9)	0.072	<0.001	2705 (69.2)	2670 (68.3)	0.019	0.393
Residence at ECMO therapy				0.604				0.945
Capital city (Seoul)	917 (23.4)	3081 (23.1)			917 (23.4)	928 (23.7)		
Other metropolitan city	885 (22.6)	3119 (23.4)	0.018		885 (22.6)	876 (22.4)	0.005	
Other area	2107 (53.9)	7128 (53.5)	0.008		2107 (53.9)	2105 (53.9)	0.001	
Annual income level at ECMO therapy				<0.001				0.572
Q1 (Lowest)	995 (25.5)	3323 (24.9)			995 (25.5)	993 (25.4)		
Q2	591 (15.1)	2551 (19.1)	0.112		591 (15.1)	631 (16.1)	0.029	
Q3	859 (22.0)	3095 (23.2)	0.030		859 (22.0)	865 (22.1)	0.004	
Q4 (Highest)	1464 (37.5)	4359 (32.7)	0.098		1464 (37.5)	1420 (36.3)	0.023	
Annual case volume of ECMO therapy				0.016				0.825
Q1: <17	1049 (26.8)	3423 (25.7)			1049 (26.8)	1035 (26.5)		
Q2: 17–36	1105 (28.3)	3545 (26.6)	0.112		1105 (28.3)	1143 (29.2)	0.022	
Q3: 37–80	1035 (26.5)	3690 (27.7)	0.030		1035 (26.5)	1022 (26.1)	0.008	
Q4: >81	720 (18.4)	2670 (20.0)	0.098		720 (18.4)	709 (18.1)	0.007	
Duration of ECMO therapy, day	5.4 (6.6)	6.5 (9.4)	0.159	<0.001	5.4 (6.6)	5.4 (6.6)	0.021	0.140
Prior NOAC use	82 (2.1)	186 (1.4)	0.049	0.002	82 (2.1)	82 (2.1)	0.016	0.489
LMWH use during hospitalization	361 (9.2)	1002 (7.5)	0.059	<0.001	361 (9.2)	361 (9.2)	0.004	0.845
Prior statin use	945 (24.2)	2847 (21.4)	0.092	<0.001	945 (24.2)	921 (23.6)	0.008	0.354
Prior warfarin use	39 (1.0)	170 (1.3)	0.030	<0.001	39 (1.0)	45 (1.2)	0.001	0.210
Charlson comorbidity index	4.7 (2.9)	4.0 (2.8)	0.236	<0.001	4.7 (2.9)	4.7 (2.9)	0.018	0.421
Hypertension, complicated	985 (25.2)	1496 (11.2)	0.321	<0.001	985 (25.2)	894 (22.9)	0.012	0.016
Hypertension, uncomplicated	3608 (92.3)	7756 (58.2)	1.279	<0.001	3608 (92.3)	3623 (92.7)	0.002	0.521
Myocardial infarction	1772 (45.3)	4121 (30.9)	0.290	<0.001	1772 (45.3)	1732 (44.3)	0.022	0.363
Congestive heart failure	1912 (48.9)	5100 (38.3)	0.213	<0.001	1912 (48.9)	1861 (47.6)	0.026	0.248
Peripheral vascular disease	1255 (32.1)	2380 (17.9)	0.305	<0.001	1255 (32.1)	1212 (31.0)	0.024	0.295
Cerebrovascular disease	1168 (29.9)	2113 (15.9)	0.306	<0.001	1168 (29.9)	1072 (27.4)	0.054	0.016
Dementia	276 (7.1)	452 (3.4)	0.142	<0.001	276 (7.1)	264 (6.8)	0.012	0.593
Chronic pulmonary disease	1631 (41.7)	5444 (40.8)	0.018	0.327	1631 (41.7)	1717 (43.9)	0.045	0.052
Rheumatic disease	243 (6.2)	907 (6.8)	0.024	0.195	243 (6.2)	250 (6.4)	0.007	0.745
Peptic ulcer disease	1291 (33.0)	4035 (30.3)	0.059	0.001	1291 (33.0)	1301 (33.3)	0.005	0.810
Mild liver disease	1412 (36.1)	4642 (34.8)	0.027	0.136	1412 (36.1)	1429 (36.6)	0.009	0.689
Diabetes without chronic complication	2022 (51.7)	6183 (46.4)	0.107	<0.001	2022 (51.7)	2049 (52.4)	0.014	0.541
Diabetes with chronic complication	924 (23.6)	1668 (12.5)	0.262	<0.001	924 (23.6)	876 (22.4)	0.029	0.197
Hemiplegia or paraplegia	118 (3.0)	298 (2.2)	0.046	0.005	118 (3.0)	114 (2.9)	0.006	0.790
Renal disease	548 (14.0)	1211 (9.1)	0.142	<0.001	548 (14.0)	541 (13.8)	0.005	0.819
Any malignancy	590 (15.1)	2493 (18.7)	0.101	<0.001	590 (15.1)	579 (14.8)	0.008	0.727
Moderate or severe liver disease	133 (3.4)	1075 (8.1)	0.257	<0.001	133 (3.4)	126 (3.2)	0.010	0.658
Metastatic solid tumor	82 (2.1)	515 (3.9)	0.123	<0.001	82 (2.1)	93 (2.4)	0.020	0.400
AIDS/HIV	2 (0.1)	26 (0.2)	0.064	0.049	2 (0.1)	1 (0.0)	0.011	0.564
Dyslipidemia	2677 (68.5)	5678 (42.6)	0.557	<0.001	2677 (68.5)	2578 (66.0)	0.055	0.018
Year of ECMO therapy				<0.001				0.535
2009–2010	293 (7.5)	1275 (9.6)			293 (7.5)	328 (8.4)		
2011–2012	687 (17.6)	2049 (15.4)	0.058		687 (17.6)	651 (16.7)	0.024	
2013–2014	877 (22.4)	2886 (21.7)	0.019		877 (22.4)	869 (22.2)	0.005	
2015–2016	917 (23.5)	3317 (24.9)	0.034		917 (23.5)	909 (23.3)	0.005	
2017–2018	1135 (29.0)	3801 (28.5)	0.011		1135 (29.0)	1152 (29.5)	0.010	
Main diagnosis at ECMO therapy				<0.001				0.402
Cardiovascular group	3134 (80.2)	7895 (59.2)			3134 (80.2)	3088 (79.0)		
Respiratory group	304 (7.8)	1699 (12.7)	0.186		304 (7.8)	330 (8.4)	0.025	
Other group	471 (12.0)	3734 (28.0)	0.490		471 (12.0)	491 (12.6)	0.016	

Data are presented as mean and standard deviation or as number and percentage. PS, propensity score; ASD, absolute value of standardized mean difference; ECMO, extracorporeal membrane oxygenation; NOAC, novel oral anticoagulant; LMWH, low-molecular-weight heparin; AIDS, acquired immune deficiency syndrome; HIV, human immunodeficiency virus.

**Table 5:** Stroke incidence before and after PS matching.

Variable	Event (%)	Logistic Regression Analysis	*p*-Value
		Odds Ratio (95% CI)	
Before PS matching			
Total stroke			
Control	608/13,328 (4.6)	1	
Anti-PLT	171/3909 (4.4)	0.96 (0.81, 1.14)	0.620
Ischemic stroke			
Control	388/13,328 (2.9)	1	
Anti-PLT	121/3909 (3.1)	1.07 (0.87, 1.31)	0.550
Hemorrhagic stroke			
Control	245/13,328 (1.8)	1	
Anti-PLT	51/3909 (1.3)	0.71 (0.52, 0.96)	0.025
After PS matching			
Total stroke			
Control	233/3909 (6.0)	1	
Anti-PLT	171/3909 (4.4)	0.75 (0.60, 0.90)	0.009
Ischemic stroke			
Control	154/3909 (3.9)	1	
Anti-PLT	121/3909 (3.1)	0.79 (0.60, 0.98)	0.043
Hemorrhagic stroke			
Control	79/3909 (1.9)	1	
Anti-PLT	51/3909 (1.3)	0.65 (0.50, 0.94)	0.020

PS, propensity score; CI, confidence interval; PLT, platelet.

## Data Availability

Data will be available upon reasonable request to corresponding author.

## Supplementary Materials

The following are available online at https://www.mdpi.com/article/10.3390/ijerph18168679/s1, Table S1. ICD-10 codes used by comorbidity to compute the Charlson comorbidity index, Table S2. Multivariable logistic regression analysis for the occurrence of stroke among patients undergoing ECMO who survived for ≥7 days (*n* = 11,975). Table S3. Multivariable logistic regression analysis for stroke within 30 days among total ECMO patients.

## References

[1] Gattinoni L., Carlesso E., Langer T. (2011). Clinical review: Extracorporeal membrane oxygenation. Crit. Care.

[2] Combes A., Hajage D., Capellier G., Demoule A., Lavoue S., Guervilly C., Da Silva D., Zafrani L., Tirot P., Veber B. (2018). Extracorporeal Membrane Oxygenation for Severe Acute Respiratory Distress Syndrome. N. Engl. J. Med..

[3] Lafc G., Budak A.B., Yener A.U., Cicek O.F. (2014). Use of extracorporeal membrane oxygenation in adults. Heart Lung Circ..

[4] Tramm R., Ilic D., Davies A.R., Pellegrino V.A., Romero L., Hodgson C. (2015). Extracorporeal membrane oxygenation for critically ill adults. Cochrane Database Syst. Rev..

[5] Eckman P.M., Katz J.N., El Banayosy A., Bohula E.A., Sun B., van Diepen S. (2019). Veno-Arterial Extracorporeal Membrane Oxygenation for Cardiogenic Shock: An Introduction for the Busy Clinician. Circulation.

[6] Brodie D., Slutsky A.S., Combes A. (2019). Extracorporeal Life Support for Adults with Respiratory Failure and Related Indications: A Review. JAMA.

[7] Aneman A., Brechot N., Brodie D., Colreavy F., Fraser J., Gomersall C., McCanny P., Moller-Sorensen P.H., Takala J., Valchanov K. (2018). Advances in critical care management of patients undergoing cardiac surgery. Intensive Care Med..

[8] Cho H.W., Song I.A., Oh T.K. (2021). Trends in extracorporeal membrane oxygenation treatment from 2005 to 2018 in South Korea. Perfusion.

[9] Murphy D.A., Hockings L.E., Andrews R.K., Aubron C., Gardiner E.E., Pellegrino V.A., Davis A.K. (2015). Extracorporeal membrane oxygenation-hemostatic complications. Transfus. Med. Rev..

[10] Parzy G., Daviet F., Puech B., Sylvestre A., Guervilly C., Porto A., Hraiech S., Chaumoitre K., Papazian L., Forel J.M. (2020). Venous Thromboembolism Events Following Venovenous Extracorporeal Membrane Oxygenation for Severe Acute Respiratory Syndrome Coronavirus 2 Based on CT Scans. Crit. Care Med..

[11] McCarthy F.H., McDermott K.M., Kini V., Gutsche J.T., Wald J.W., Xie D., Szeto W.Y., Bermudez C.A., Atluri P., Acker M.A. (2015). Trends in U.S. Extracorporeal Membrane Oxygenation Use and Outcomes: 2002–2012. Semin. Thorac. Cardiovasc. Surg..

[12] Millar J.E., Fanning J.P., McDonald C.I., McAuley D.F., Fraser J.F. (2016). The inflammatory response to extracorporeal membrane oxygenation (ECMO): A review of the pathophysiology. Crit. Care.

[13] Le Guennec L., Cholet C., Huang F., Schmidt M., Brechot N., Hekimian G., Besset S., Lebreton G., Nieszkowska A., Leprince P. (2018). Ischemic and hemorrhagic brain injury during venoarterial-extracorporeal membrane oxygenation. Ann. Intensive Care.

[14] Omar H.R., Mirsaeidi M., Shumac J., Enten G., Mangar D., Camporesi E.M. (2016). Incidence and predictors of ischemic cerebrovascular stroke among patients on extracorporeal membrane oxygenation support. J. Crit. Care.

[15] Saeed O., Jakobleff W.A., Forest S.J., Chinnadurai T., Mellas N., Rangasamy S., Xia Y., Madan S., Acharya P., Algodi M. (2019). Hemolysis and Nonhemorrhagic Stroke during Venoarterial Extracorporeal Membrane Oxygenation. Ann. Thorac. Surg..

[16] Aubron C., DePuydt J., Belon F., Bailey M., Schmidt M., Sheldrake J., Murphy D., Scheinkestel C., Cooper D.J., Capellier G. (2016). Predictive factors of bleeding events in adults undergoing extracorporeal membrane oxygenation. Ann. Intensive Care.

[17] Xie A., Lo P., Yan T.D., Forrest P. (2017). Neurologic Complications of Extracorporeal Membrane Oxygenation: A Review. J. Cardiothorac. Vasc. Anesth..

[18] Tendera M., Wojakowski W. (2003). Role of antiplatelet drugs in the prevention of cardiovascular events. Thromb. Res..

[19] Oprea A.D., Popescu W.M. (2013). Perioperative management of antiplatelet therapy. Br. J. Anaesth..

[20] Von Elm E., Altman D.G., Egger M., Pocock S.J., Gotzsche P.C., Vandenbroucke J.P., Initiative S. (2014). The Strengthening the Reporting of Observational Studies in Epidemiology (STROBE) Statement: Guidelines for reporting observational studies. Int. J. Surg..

[21] Barbaro R.P., Odetola F.O., Kidwell K.M., Paden M.L., Bartlett R.H., Davis M.M., Annich G.M. (2015). Association of hospital-level volume of extracorporeal membrane oxygenation cases and mortality. Analysis of the extracorporeal life support organization registry. Am. J. Respir. Crit. Care Med..

[22] Rosenbaum P.R., Rubin D.B. (1983). The central role of the propensity score in observational studies for causal effects. Biometrika.

[23] Malfertheiner M.V., Koch A., Fisser C., Millar J.E., Maier L.S., Zeman F., Poschenrieder F., Lubnow M., Philipp A., Muller T. (2020). Incidence of early intra-cranial bleeding and ischaemia in adult veno-arterial extracorporeal membrane oxygenation and extracorporeal cardiopulmonary resuscitation patients: A retrospective analysis of risk factors. Perfusion.

[24] Fletcher-Sandersjoo A., Bartek J., Thelin E.P., Eriksson A., Elmi-Terander A., Broman M., Bellander B.M. (2017). Predictors of intracranial hemorrhage in adult patients on extracorporeal membrane oxygenation: An observational cohort study. J. Intensive Care.

[25] Kasirajan V., Smedira N.G., McCarthy J.F., Casselman F., Boparai N., McCarthy P.M. (1999). Risk factors for intracranial hemorrhage in adults on extracorporeal membrane oxygenation. Eur. J. Cardiothorac. Surg..

[26] Koster A., Ljajikj E., Faraoni D. (2019). Traditional and non-traditional anticoagulation management during extracorporeal membrane oxygenation. Ann. Cardiothorac. Surg..

[27] Cheng W., Ma X.D., Su L.X., He H.W., Wang L., Tang B., Du W., Zhou Y.K., Wang H., Cui N. (2020). Cross-sectional study for the clinical application of extracorporeal membrane oxygenation in Mainland China, 2018. Crit. Care.

[28] Schmidt M., Hajage D., Lebreton G., Monsel A., Voiriot G., Levy D., Baron E., Beurton A., Chommeloux J., Meng P. (2020). Extracorporeal membrane oxygenation for severe acute respiratory distress syndrome associated with COVID-19: A retrospective cohort study. Lancet Respir. Med..

[29] Iacobelli R., Fletcher-Sandersjoo A., Lindblad C., Keselman B., Thelin E.P., Broman L.M. (2021). Predictors of brain infarction in adult patients on extracorporeal membrane oxygenation: An observational cohort study. Sci. Rep..

[30] Shrestha S., Coy S., Bekelis K. (2017). Oral Antiplatelet and Anticoagulant Agents in the Prevention and Management of Ischemic Stroke. Curr. Pharm. Des..

[31] Geisler T., Poli S., Meisner C., Schreieck J., Zuern C.S., Nagele T., Brachmann J., Jung W., Gahn G., Schmid E. (2017). Apixaban for treatment of embolic stroke of undetermined source (ATTICUS randomized trial): Rationale and study design. Int. J. Stroke.

[32] Serebruany V.L., Malinin A.I., Eisert R.M., Sane D.C. (2004). Risk of bleeding complications with antiplatelet agents: Meta-analysis of 338,191 patients enrolled in 50 randomized controlled trials. Am. J. Hematol..

[33] Luyt C.-E., Bréchot N., Demondion P., Jovanovic T., Hékimian G., Lebreton G., Nieszkowska A., Schmidt M., Trouillet J.-L., Leprince P.J.I.c.m. (2016). Brain injury during venovenous extracorporeal membrane oxygenation. Intensive Care Med..

[34] Chen C., Wang X., Chen X., Ouyang M., Sun L., Arima H., Robinson T., Lindley R.I., Chalmers J., Li G. (2021). Disparities between Asian and Non-Asian Thrombolyzed Acute Ischemic Stroke Patients in the Enhanced Control of Hypertension and Thrombolysis Stroke Trial. Cerebrovasc. Dis..

