# Supplementary material for: Prior Antiplatelet Therapy and Stroke Risk in Critically Ill Patients Undergoing Extracorporeal Membrane Oxygenation
Source: Int J Environ Res Public Health. 2021 Aug 17;18(16):8679. doi: 10.3390/ijerph18168679 (PMC8394632; doi:10.3390/ijerph18168679)
Supplement: Supplementary file 1 [file ijerph-18-08679-s001.zip › Table S2.pdf]

Table S2. Multivariable logistic regression analysis for occurrence of stroke among ECMO patients who lived lived ≥ 7 days (n=11,975)

Variable	Multivariable model<br>OR (95% CI)	*P*-value
Total stroke		
Anti-PLT group (vs control)	0.74 (0.58, 0.95)	0.026
Ischemic stroke		
Anti-PLT group (vs control)	0.78 (0.59, 0.99)	0.048
Hemorrhagic stroke		
Anti-PLT group (vs control)	0.75 (0.52, 1.21)	0.238

OR, odds ratio; CI, confidence interval
