# Supplementary material for: Prior Antiplatelet Therapy and Stroke Risk in Critically Ill Patients Undergoing Extracorporeal Membrane Oxygenation
Source: Int J Environ Res Public Health. 2021 Aug 17;18(16):8679. doi: 10.3390/ijerph18168679 (PMC8394632; doi:10.3390/ijerph18168679)
Supplement: Supplementary file 1 [file ijerph-18-08679-s001.zip › Table S3.pdf]

Table S3. Multivariable logistic regression analysis for stroke within 30 days among total ECMO patients

Variable	Multivariable model<br>OR (95% CI)	*P*-value
Total stroke		
Anti-PLT group (vs control)	0.70 (0.57, 0.85)	<0.001
Ischemic stroke		
Anti-PLT group (vs control)	0.75 (0.62, 0.94)	0.009
Hemorrhagic stroke		
Anti-PLT group (vs control)	0.60 (0.42, 0.83)	0.003

OR, odds ratio; CI, confidence interval
